# Wastewater treatment plants as a source of microplastics in river catchments

**DOI:** 10.1007/s11356-018-2070-7

**Published:** 2018-06-07

**Authors:** Paul Kay, Robert Hiscoe, Isobel Moberley, Luke Bajic, Niamh McKenna

**Affiliations:** School of Geography/water@leeds, University of Leeds, Leeds, West Yorkshire LS2 9JT UK

**Keywords:** Microplastics, Emerging contaminants, Rivers, Water quality, Pollution, Wastewater

## Abstract

It is now well established that the oceans contain significant accumulations of plastic debris but only very recently have studies began to look at sources of microplastics (MPs) in river catchments. This work measured MPs up- and downstream of six wastewater treatment plants (WWTPs) in different catchments with varying characteristics and found that all led to an increase in MPs in rivers. Nevertheless, the data collected indicated that there were other important sources of MPs in the catchments studied and that these may include atmospheric deposition, agricultural land to which sewage sludge has been applied, and diffuse release of secondary MPs following the breakdown of larger plastic items. MPs were comprised mainly of fibres, fragments, and flakes with pellets and beads only dominating at one site. Variation in MP pollution occurred over time and this difference was greater at some sites than others. A key research need is the further study of MP sources in river catchments to facilitate management efforts to reduce their presence in freshwater and marine environments.

## Introduction

The ‘plastic age’ comes with significant benefits but also problems, including the accumulation of microplastics (MPs) in the aquatic environment (Wagner et al. 2014). MPs are one of the least studied groups of emerging contaminants in river systems (Blair et al. 2017) despite the fact that most of those in the marine environment are thought to derive from terrestrial environments and to have been transported in fluvial systems (Blair et al. 2017). Various reasons have been postulated as to why research has focused almost entirely on the marine environment, including MPs being more visible on oceans and beaches and effects having been observed in marine biota (Ryan et al. 2009). MPs are defined as pieces of plastic with a diameter < 5 mm and can come from a diverse range of sources, including personal care products, textiles, and packaging. The effects of MPs entering the environment include their ingestion by organisms, ranging from invertebrates to fish, which may have direct impacts as well as expose these organisms to pollutants attached to the MPs (Wagner et al. 2014; Eerkes-Medrano et al. 2015). It has been postulated that wastewater treatment plants (WWTPs) are the main source of MPs to river catchments (Roex et al. 2013) as they receive waste from industries manufacturing and using MPs, for instance as scrubbers in cleaning and cosmetic products, as well as domestic effluent from households using products containing MPs (Zbyszewski et al. 2014; Eerkes-Medrano et al. 2015). Furthermore, secondary MPs may result from the breakdown of plastic products used in river catchments, such as those used by consumers and in industry (Blair et al. 2017). But, most studies of MPs in freshwater environments have focused on large lakes (e.g. Zbyszewski and Corcoran 2011; Zbyszewski et al. 2014; Ballent et al. 2016) rather than rivers which receive WWTP effluent. In the few available papers, it has been observed that MP concentrations may be elevated downstream of WWTPs (McCormick et al. 2014; Morritt et al. 2014; Estahbanati and Fahrenfeld 2016) but that, conversely, no increase may occur through urban areas (Dris et al. 2015). Relatively unpopulated areas of catchments may not be a source of MPs (Sadri and Thompson 2014). Recent research has highlighted that one of the most important needs in this new area of water research is to study MPs in rivers, particularly those receiving effluent from WWTPs (Wagner et al. 2014; Eerkes-Medrano et al. 2015; Blair et al. 2017). Specific objectives were to:

- Understand the contribution of WWTPs to the microplastic loading of rivers;
- Determine the composition of MPs in rivers up- and downstream of WWTPs.

## Methods

### Field sites

Field sites were selected across the north of England to have a variety of characteristics in terms of the population equivalent (PE) served by the WWTPs, treatment technologies used, and catchment characteristics (Table 1). This allowed a broad understanding to be obtained of how these factors determine the extent to which WWTPs contribute to MP pollution of receiving waters.

**Table 1:** Description of wastewater treatment plants (WWTPs) that were sampled

WWTP	River catchment	Population equivalent served (K)	Treatment technology	Upstream catchment land use	Grid reference
1 Wanlip	Soar	900	Activated sludge	Urban, agriculture, no WWTP	SK598191163
2 Barnard Castle	Tees	10	Trickling filter	Urban, agriculture, WWTP	Z0591415407
3 Horbury Junction	Calder	16	Secondary biological filter	Urban, agriculture, WWTP	SE3006917238
4 Naburn	Yorkshire Ouse	237	Activated sludge	Urban, agriculture, WWTP	SE6005446754
5 Driffield	Hull	145	Activated sludge	Agriculture, no WWTP	TA0289456856
6 Thorp Arch	Wharfe	10	Secondary biological filter	Urban, agriculture, WWTP	SE4503445874

### Sampling

There are currently no standard accepted methods for the monitoring of MPs in rivers (Blair et al. 2017) but, as done in most other studies of MPs in the aquatic environment (Hidalgo-Ruz et al. 2012), a 300-μm mesh size net was used. The net frame measured 250 mm by 230 mm and was attached to a wooden pole which was used to hold the net in the water on each sampling occasion for 15 min. The frame of the net was held against the bed of the river, facing upstream, and MPs were rinsed from the net into a sorting tray using deionised water and then transferred to a sample bottle for storage. Samples were refrigerated at 4 °C to limit bacterial growth before identification of MPs with 48 h. Five replicate samples were collected at each site over a 6-week period, other than site one where three replicates were collected.

### Laboratory analysis

Samples were passed through a series of six 20-cm-diameter steel stacked sieves of mesh sizes 5.6 mm, 4 mm, 2 mm, 1 mm, 500 μm, and 250 μm, to allow visual sorting and identification of solid material (Hidalgo-Ruz et al. 2012). After a sample was poured through the sieves, the sample bottle and lid were rinsed three times each with deionised water which was also filtered to ensure no material remained in the bottle. Deionised water was then run through the sieves and these were shaken to ensure all particulate matter was caught on the appropriate size sieve; this process was repeated three times. Any material from the top sieve (5.6 mm) was discarded. Any organic matter was identified visually and removed and MPs transferred to a petri dish using deionised water. Tweezers were used to transfer any MPs to the petri dish that were not displaced by the deionised water. Sieves were thoroughly rinsed using a pressurised tap before the next sample was passed through. MPs in each petri dish were then examined under a stereomicroscope (Brunel Microscopes Ltd, UK). Plastics were identified based on the fact that they were homogenous particles, with no obvious cellular structure, and could easily be dented with tweezers but not broken apart (Mani et al. 2015), other than for plastic foam (McCormick et al. 2014). They were categorised into pellets/beads, fibres, and fragments/flakes.

## Results

### Contribution of wastewater treatment plants to microplastic loading of receiving waters

**Fig. 1:**
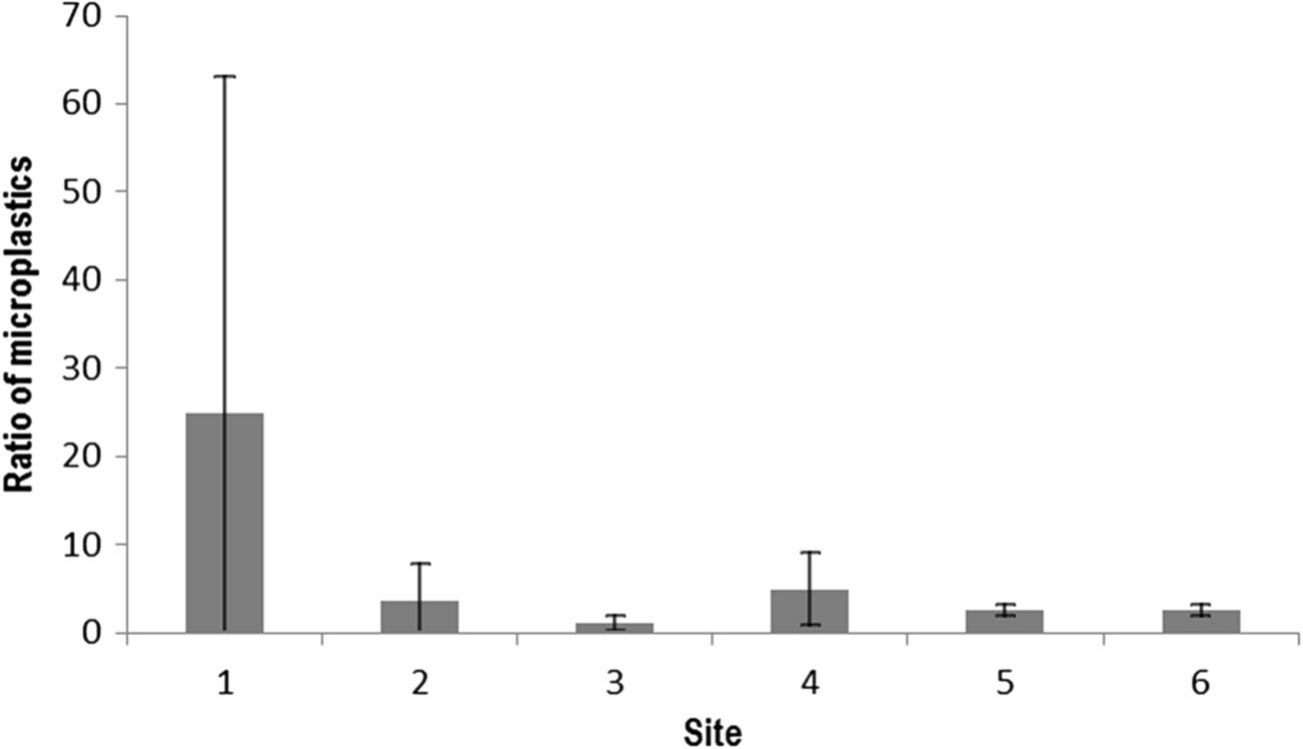
Ratio of microplastics up- and downstream of wastewater treatment plants (WWTPs). A positive ratio indicates an increase due to the WWTP. *n* = 3 at site 1 and 5 at all other sites. Error bars indicate one standard deviation. See Table 1 for details of sampling sites

The quantity of microplastics generally increased downstream of each WWTP monitored with the mean ratio up- and downstream always being greater than 1 (Fig. 1). Ratios were typically between 1 and 3 (for 19 out of 28 paired samples).

Nevertheless, on 4 occasions out of 28, MPs were found to be higher upstream, caused by a combination of increases upstream and decreases downstream. There was little temporal variability at some sites but more at others. This was most substantial at site 1 due to one particular sampling event where the number of microplastics found downstream was 69 times higher than upstream.

### Composition of microplastics up- and downstream of wastewater treatment plants

**Fig. 2:**
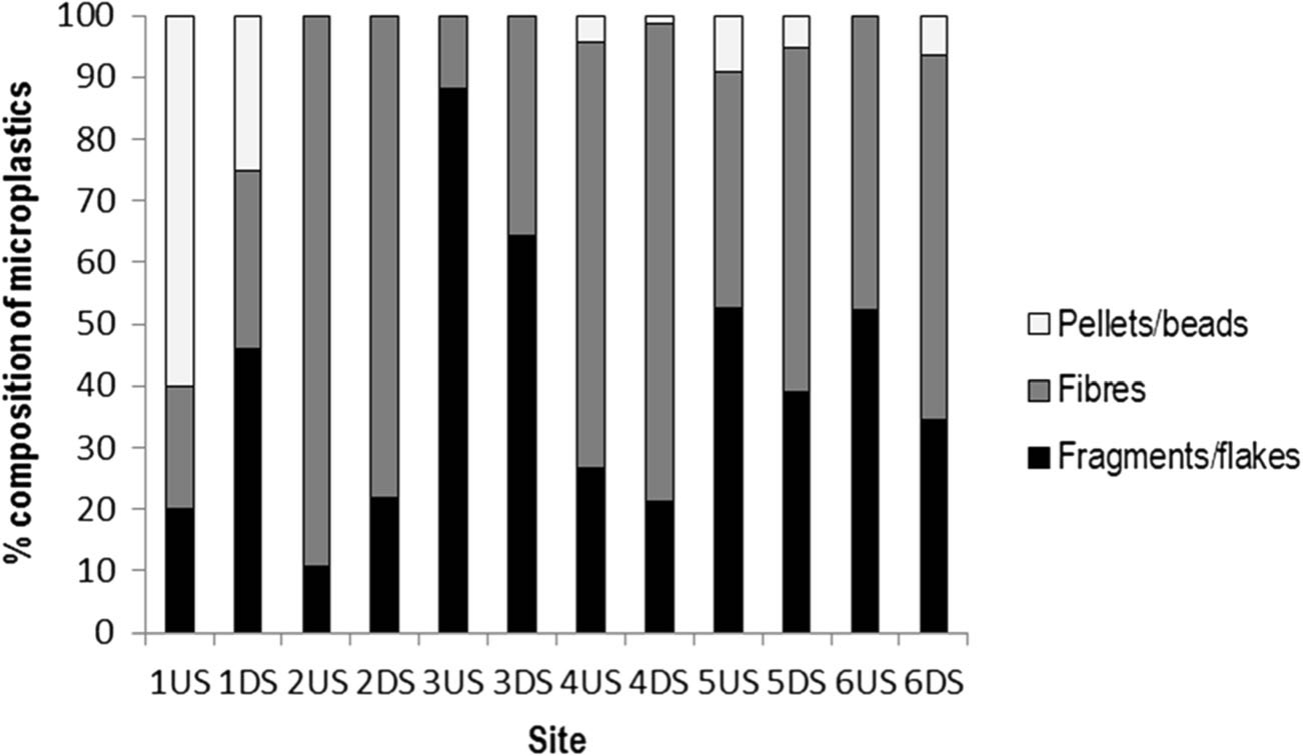
Mean composition of microplastics in receiving waters up- (US) and downstream (DS) of wastewater treatment plants. *n* = 3 at site 1 and 5 at all other sites. See Table 1 for details of sampling sites

The composition of MPs both up- and downstream of WWTPs comprised mainly of fragments and fibres with relatively few pellets and beads, the latter usually making up less than 10% of the total (Fig. 2) other than for site 1 where the percentage was heavily skewed due to some beads being found amongst a small total number of MPs.

## Discussion

### Sources of microplastics in river catchments

The fact that the quantity of microplastics present in receiving waters was greater downstream of each of the six WWTPs studied confirms that treated sewage effluent is a key source of MPs, agreeing with the very limited number of other available studies (McCormick et al. 2014; Morritt et al. 2014; Estahbanati and Fahrenfeld 2016). Nevertheless, there was variation in the extent of pollution from individual works over time, as has been observed by others (Magnusson and Norén 2014; Estahbanati and Fahrenfeld 2016) and there was a range of variation at different WWTPs. There was a strong relationship between the population equivalent served and the amount of variation which existed over time although this is driven by the much higher PE served by the Wanlip works and the relationship does not hold if this site is removed from the data set. Further work is therefore needed to elucidate the cause of this variation. Nevertheless, MPs were also found upstream of all of the WWTPs (Fig. 2). At some sites, this could potentially be attributed to WWTPs further upstream but at sites 1 and 5, there were no WWTPs upstream; indeed, at site 5, less than 1 km of stream exists upstream of the WWTP and the catchment is agricultural. This indicates that significant sources of MPs other than WWTPs exist in river catchments, agreeing with previous work (Dris et al. 2015; Estahbanati and Fahrenfeld 2016), which may comprise agricultural land to which sewage sludge has been applied, breakdown of plastics used in agriculture, and aerial deposition of MPs from other areas. Currently, minimal literature exists on sources of microplastics in river catchments and this is a key research need.

### Composition of microplastics in river catchments

A consistent pattern in MP composition did not exist across all sites although fibres, fragments, and flakes usually dominated, consistent with some previous work (Dris et al. 2015; Ballent et al. 2016), and beads and pellets were only dominant at the site 1 upstream sampling location, as for sites sampled by Mani et al. (2015). This suggests that the sources of MPs in river catchments are diverse and may vary within and across catchments and that MP source apportionment work is needed. The prevalence of fibres at many sites indicates that the breakdown of textiles is a key source of MPs whilst the considerable presence of fragments and flakes suggests that secondary MPs are also very important and efforts to reduce MPs in rivers and oceans need to focus on sources of these types of MPs.

## Conclusions

This study was one of the first to measure microplastics in river catchments and determine potential sources of pollution. WWTPs are key sources of MPs in river catchments although others are also clearly important and these may include sewage sludge applied to agricultural land, diffuse release of secondary MPs, and aerial deposition. MP composition varies spatially and temporally but is dominated by fibres, fragments, and flakes as opposed to beads and pellets. Management efforts to reduce MP concentrations in rivers and oceans must focus on a diverse range of MP sources.
